# Quantitative study on the environmental impact of Beijing’s urban rail transit based on carbon emission reduction

**DOI:** 10.1038/s41598-025-86714-4

**Published:** 2025-01-18

**Authors:** Cai Jia, Xudong Wang, Chengyang Qian, Zini Cao, Long Zhao, Luzhou Lin

**Affiliations:** 1 School of Geography and Tourism, Anhui Normal University, Huajin Campus, South 189 Jiuhua Rd, Wuhu, 241002 China; 2 Engineering Technology Research Center of Resources Environment and GIS, Wuhu, 241008 China; 3 Academy of Regional and Global Governance, Beijing Foreign Studies University, Beijing, 100089 China; 4 SIPSG Information Technology Co. Ltd., Suzhou, 215004 China

**Keywords:** Climate-change mitigation, Energy and society, Sustainability

## Abstract

Urban rail transit, as an efficient and eco-friendly mode of transportation, plays a pivotal role in mitigating traffic congestion and lowering urban carbon emissions. Despite the significant contributions by scholars in this area, debates surrounding the quantification of carbon emissions during the operational phase of urban rail transit persist, particularly in assessing its impact on reducing ground traffic congestion. This study examines the passenger flow during Beijing’s morning and evening peak hours, assuming that all passengers initially using urban rail transit switch to buses and taxis during these periods. A traffic congestion prediction model is developed based on the analysis of actual traffic operation data under this assumption. Through this model, the study calculates the potential congestion times across various scenarios, employing a bottom-up approach to carbon emission estimation to analyze the impact on carbon emissions. Results spanning 2015 to 2021 suggest that substituting urban rail transit with buses could increase congestion by 37–92 min and 46–59 min during morning and evening peaks, respectively, leading to a 24-82% and 27-56% surge in carbon emissions. The conversion of all these vehicles to taxis would result in a direct paralysis of Beijing’s road transport network, with a corresponding increase in carbon emissions of between 289% and 556% and 333% and 614%, respectively.These outcomes emphasize the substantial efficacy of urban rail transit in curbing traffic congestion and carbon emissions.

## Introduction

As the Central Committee of the Communist Party of China and the State Council advance their work on carbon peaking and neutrality, the concurrent reduction of carbon emissions and alleviation of traffic congestion has emerged as a principal challenge in China’s transportation domain^1,2^. Despite the implementation of low-carbon development strategies focused on green travel in major cities, carbon emissions from the transport sector continue to contribute over 10% to the national total. Road transport alone accounts for approximately 80% of these emissions and is increasing^3–5^. Consequently, urban rail transit, as a public transportation mode characterized by low emissions and high energy efficiency, is receiving increasing attention^6^. By the end of 2022, 55 cities in China had inaugurated urban rail transit operations, amassing a total route length of 10,287.45 km, significantly contributing to the mitigation of urban traffic congestion and the reduction of carbon emissions^7,8^.

As the capital of China and an international metropolis with a population of 21,843,000 (as of 2022), Beijing has experienced rapid economic development, which has led to a significant increase in carbon emissions from the transport sector. Statistical data indicates that motor vehicle emissions constitute approximately 60% of the city’s air pollution, representing the primary source of contamination^9^. This pollution represents a significant threat to the health and well-being of residents, as well as an obstacle to the sustainable development of the city’s economy. In this context, the issue of urban traffic congestion is particularly prevalent, particularly during the morning and evening peak periods, when the pressure of commuting is considerable, and the rapid growth of private automobiles has contributed to an increase in fuel consumption and greenhouse gas emissions^10^. Furthermore, Beijing is among the most congested cities in China, with the highest per capita cost of congestion exceeding RMB 8,000^11^. This is a significant challenge to urban development, as increased energy consumption and pollutant emissions due to congestion are major constraints on the city’s growth.

As an efficient mode of public transportation, rail transit plays a vital role in alleviating surface traffic pressure, reducing pollutant emissions, and enhancing commuting efficiency. However, despite the continuous expansion of rail networks, high passenger volumes during morning and evening peak hours still strain the transport system, leaving congestion on some surface roads unresolved. This affects overall travel efficiency and indirectly impacts energy consumption and emissions control. Therefore, studying the actual effects of rail transit in mitigating traffic congestion and reducing carbon emissions holds significant practical value.

In light of the aforementioned background, this study focuses on two central questions:

1. What has been the trend in carbon emissions from Beijing’s public transport system during morning and evening peak periods over the past few years?
2. To what extent can urban rail transit contribute to alleviating traffic congestion and reducing carbon emissions?

This study utilizes traffic operation data from Beijing, focusing on passenger flow during morning and evening peak hours. It assumes that the rail transit passenger flow is fully transferred to ground buses or taxis during these periods. A traffic congestion prediction model is applied to simulate and analyze the resulting changes in congestion time. Furthermore, a bottom-up carbon emission calculation method is used to develop a carbon emission reduction assessment model for rail transit, aiming to quantify the reduction in carbon dioxide emissions attributable to its operation. This research framework provides a scientific basis for quantitatively assessing the impact of rail transit on surface traffic congestion and carbon emissions, while offering theoretical support for the formulation and optimization of related policies.

This study contributes to the field by systematically assessing the effectiveness of rail transit in mitigating traffic congestion and reducing carbon emissions. Using Beijing’s morning and evening peak hours as a case study, it highlights a pivotal time frame often overlooked in previous research. Unlike prior studies that primarily focus on the transportation sector as a whole or annual carbon emission levels, this study emphasizes peak-hour analysis, addressing a critical gap in quantitative research on the environmental benefits of rail transit during peak hours in China. The subsequent literature review will elaborate on the research gaps identified in related studies.

The rest of the paper is structured as follows: Sect. 2 reviews the relevant literature. Section 3 describes the methodologies and data, including those related to traffic congestion prediction, carbon emission measurement, carbon emission reduction assessment, and data specifics. Section 4 presents the study’s results, with Sect. 4.1 analyzing congestion during morning and evening peaks under different scenarios in Beijing, Sect. 4.2 reporting carbon emissions, and Sect. 4.3 examining the contribution of urban rail transit to carbon emission reduction during peak hours. Finally, Sect. 5 summarizes the key findings and provides policy recommendations.

## Literature review

In recent years, domestic and international scholars have extensively studied the carbon emission impacts of urban rail transit to evaluate its low-carbon environmental benefits. The research primarily focuses on three aspects: (1) *Carbon emission measurement of urban rail transit*: Researchers have developed carbon emission reduction assessment models^12–14^ by analyzing changes in travel modes before and after the opening of individual subway lines^15,16^. Using life cycle evaluation, they have quantified the carbon emissions and reduction potential of urban rail transit during its operational phase^17,18^. Additionally, theoretical models for carbon recovery periods have been constructed as quantitative indicators to assess the carbon emission reduction effects of urban rail transit^19^. (2) *Comparison of public transport carbon emission efficiency*: Using methods such as the super-efficiency SBM model and directional distance function, researchers have constructed GML^20^ and ML^21^ indices to analyze the efficiency and dynamic changes of carbon emissions in rail transport. These findings provide a basis for comparing the low-carbon efficiency of different public transport modes^22,23^. (3) *Decarbonization through passenger structure optimization*: Increasing the proportion of trips made by rail while reducing reliance on taxis and private cars has been shown to effectively alleviate road congestion and lower carbon emissions^23–27^. However, research specifically targeting the morning and evening peak hours remains limited, particularly in exploring the relationship between traffic characteristics and carbon emissions during these periods.

Concurrently, research on traffic congestion primarily focuses on the following aspects: (1) *The adverse effects of traffic congestion*: Studies have shown that traffic congestion reduces transport efficiency, prolongs commuting times, and increases traffic management costs. It also exacerbates pollutant emissions and significantly raises environmental costs^28,29^. (2) *Traffic congestion assessment*: Researchers have developed various methods to systematically evaluate congestion in urban road networks. These include the speed performance index, road section congestion index, and network congestion index, which incorporates traffic flow, road capacity, travel speed, and socio-economic factors. The data obtained provides a theoretical foundation for policy-making^30–32^. (3) *The role of public transport in alleviating congestion*: Relevant studies have explored the critical role of public transport network expansion in reducing traffic congestion. For example, some researchers have used models to analyze the spatial impact of Melbourne’s public transport on congestion at different times of the day and across various regions^33^. Additionally, studies have highlighted the potential of light rail systems to reduce congestion along travel corridors by diverting passenger flows^34^.

Although scholars have extensively studied the environmental effects of urban rail transit from multiple perspectives and achieved significant results^35–37^, current research still has certain shortcomings. Firstly, there is a lack of clarity in the definition of quantitative indicators for the evaluation of the environmental effects of urban rail transport, and studies are often limited to single lines or multiple transfer lines, lacking a comprehensive consideration of the entire urban rail system. Secondly, research seldom considers peak morning and evening hours, leading to an inadequate overall consideration of passenger flow during complex time periods. Additionally, existing studies tend to simplify urban rail transit as a tool for carbon reduction without fully considering its key role in alleviating traffic congestion, lacking an in-depth analysis of the mechanisms by which urban rail transit mitigates traffic congestion. Furthermore, there is a scarcity of research modeling the carbon emissions resulting from increased commuting time due to traffic congestion.

To address the aforementioned challenges, this study introduces the following innovations:

1. Comprehensive collection and analysis of Beijing’s public transport operation data, with refined measurement of carbon emissions. The study evaluates the carbon emissions of various public transport modes (including buses and taxis) in recent years, addressing limitations in the existing literature regarding data granularity and measurement precision.
2. A quantitative model is developed to assess the role of rail transit in alleviating traffic congestion and reducing carbon emissions during the morning and evening peak periods. The study examines the actual contribution of rail transit under different scenarios, providing a deeper understanding of peak-hour traffic characteristics and environmental impacts.
3. From the perspective of traffic congestion alleviation, the study conducts a detailed analysis of the mechanisms by which rail transit reduces carbon emissions, with particular emphasis on emissions resulting from extended commuting times caused by congestion. This analysis offers a more robust theoretical basis for the formulation of policies aimed at decarbonizing urban transport systems.

## Data and methodology

### Overview of the study area

**Fig. 1:**
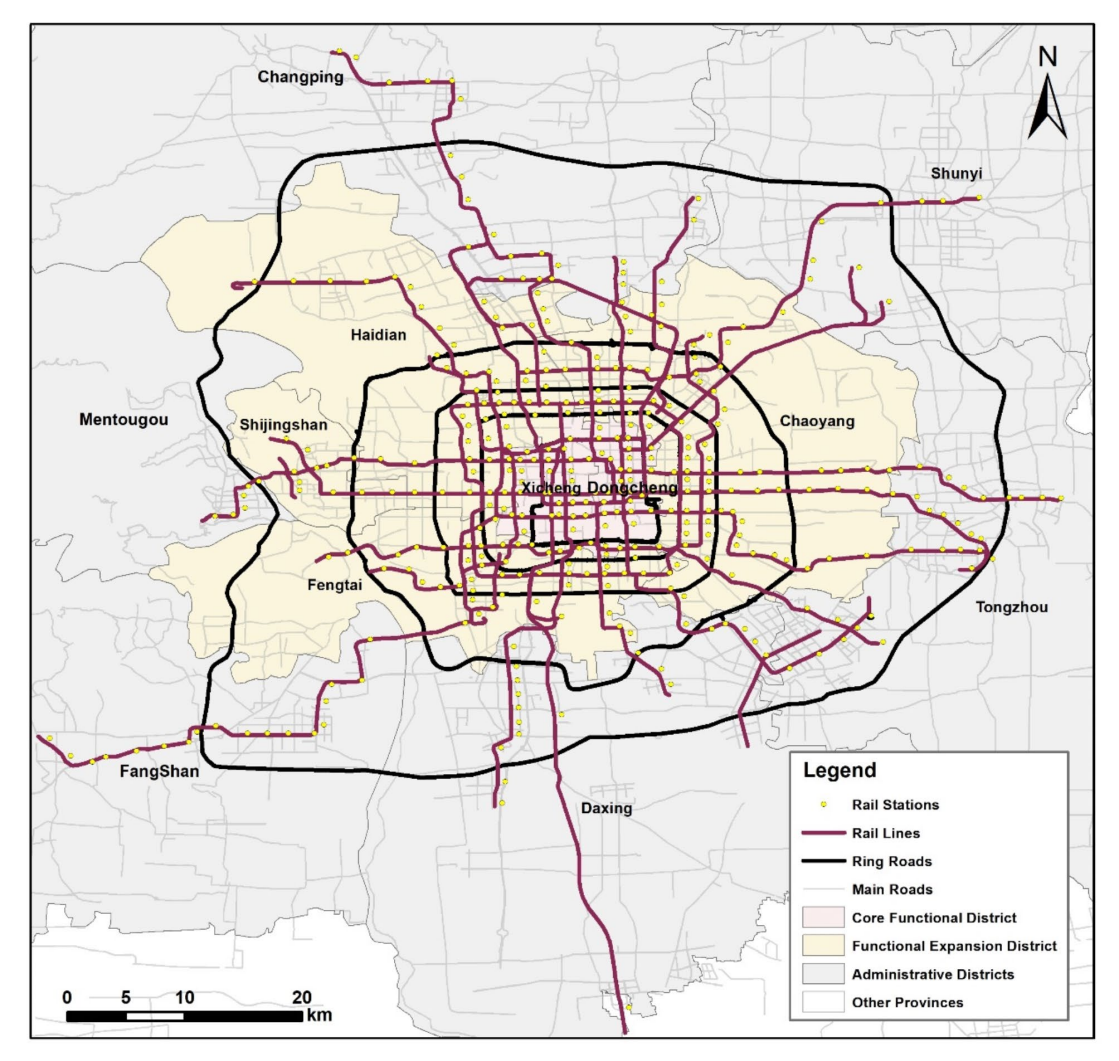
Beijing rail transit lines.

The Beijing Subway, which was constructed as the nation’s first urban rail transit system, also boasts the most lines and the second-longest operational distance of any Chinese rail transit network. It has an extensive network of 27 lines, including 22 subway lines, one medium-low speed maglev line, two modern tram lines, and two airport lines. The system spans 868 km and has 474 stations, including 62 transfer stations. Additionally, the volume of passengers utilising the Beijing Metro is on the rise, with figures surging from 2,454 million in 2012 to 3,962 million in 2019, with a record-breaking 13,753,800 passengers on a single day. Due to the COVID-19, passenger traffic decreased marginally from 2020 to 2022, but remained above 6 million passengers per day. Figure 1 depicts the subway map.

### Data sources and preprocessing

The data used in this study were obtained from the *Beijing Statistical Yearbook*^38^, the *Annual Statistical Analysis Report on Urban Rail Transport*^39^, the *Beijing Transport Development Annual Report*^40^, and the *Traffic Analysis of Major Cities in China*^41^. These sources include information on passenger traffic volume, the number of passengers transported by public buses, urban rail transit, and taxis, passenger turnover, the ratio of morning and evening peak-hour trips to total daily travel volume for different transport modes, and morning and evening peak congestion times. Additionally, the annual electricity consumption data for Beijing’s urban rail transit were sourced from the *Annual Statistical Analysis Report on Urban Rail Transport*. Detailed information about the data sources is provided in Table 1.

**Table 1:** Information on data source parameters.

Data name	Data source
Passenger volume of buses (10,000 individuals)	Beijing Statistical Yearbook
Passenger volume of urban rail transit (10,000 individuals)	Annual Statistical Analysis Report on Urban Rail Transport
Passenger volume of taxi (10,000 individuals)	Beijing Statistical Yearbook
Road passenger turnover (10,000 person-kilometres)	Beijing Statistical Yearbook
The percentage of the total daily travel volume attributed to different transportation modes during morning and evening peak hours (%)	Beijing Transport Development Annual Report
Morning and evening peak congestion time (minutes)	Traffic Analysis of Major Cities in China
Annual electricity consumption of urban rail transport (kWh)	Annual Statistical Analysis Report on Urban Rail Transport

This paper analyses statistical data on the number of buses and taxis, categorising them by model and fuel type, based on the *Annual Report on the Development of Transport in Beijing*. These data are supplemented with relevant literature^42–45^. To address discrepancies in data from Beijing Public Transport Group Co., Ltd., a weighting methodology is applied to account for variations in vehicle ownership. Using 2015 as a baseline and incorporating the latest policies^11,46^, projections are made to estimate the number of vehicles in subsequent years. Recognising the crucial role of fuel type in evaluating carbon emissions, this study examines the proportions of diesel, natural gas, and hybrid fuels used in buses and taxis. This analysis enables a more accurate assessment of transport-related carbon emissions and highlights the impact of new energy policies on vehicle structure optimisation and emission control. Specific statistics on vehicle numbers and fuel types are detailed in Table 2.

**Table 2:** Number of buses and taxis of different fuel types in Beijing.

Vehicle type	Fuel type	Year						
		2015	2016	2017	2018	2019	2020	2021
Bus	Diesel	13,797	12,089	10,381	8673	6965	5257	3549
	CNG	5564	5099	8169	6755	5823	6895	6160
	Electricity	2356	3300	4244	5189	6133	7078	8022
	Hybrid power	1570	2200	2830	3459	4089	4718	5348
Taxi	Gasoline	61,456	54,787	48,624	47,677	46,378	45,890	45,657
	CNG	1502	3424	6164	6807	7508	8489	9747
	Electricity	3004	4109	6711	7550	8480	9767	11,421
	Hybrid power	2322	6164	6985	8001	9151	10,730	12,776

**Fig. 2:**
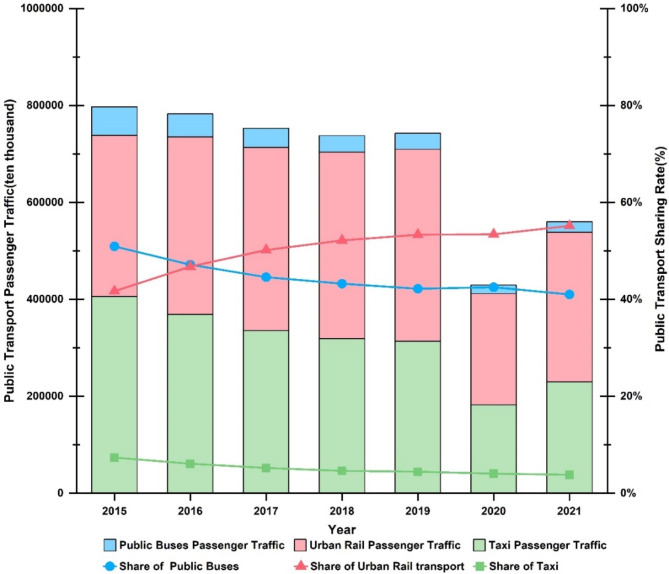
Beijing’s public transportation passenger volume and share rate.

The *Beijing Municipal Statistical Yearbook* provides data on passenger traffic volume and turnover for various modes of transport in the city. By calculating the passenger traffic volume for the three main modes, we can determine the share of journeys made by each mode. Figure 2 shows that from 2015 to 2019, both the passenger traffic volume and market share of Beijing’s urban rail transit system increased annually, while the number of journeys made by public buses (trams) and taxis steadily declined.

**Fig. 3:**
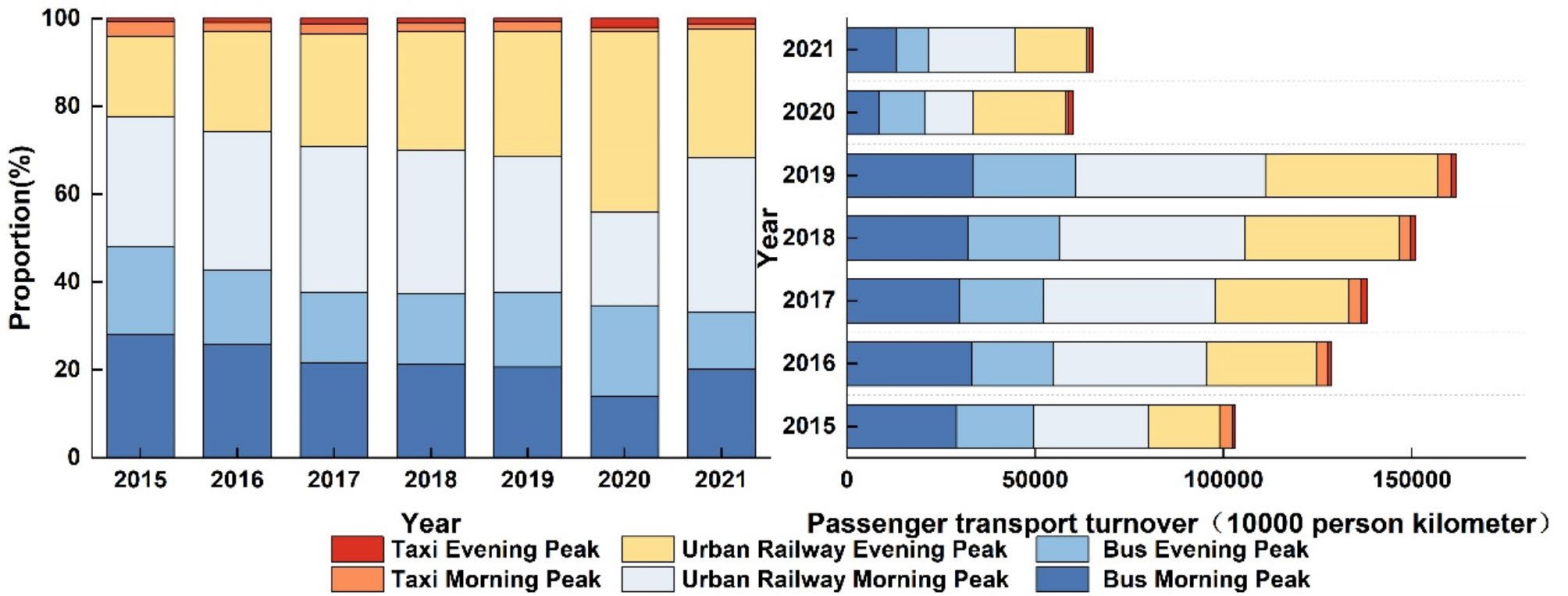
Beijing public transportation passenger turnover and proportion in the morning and evening peaks.

By using the share ratio and passenger traffic turnover of urban rail transport, the traffic turnover of the three modes can be indirectly derived. The results are then multiplied by the ratio of morning and evening peak hour trips to total daily trips for each mode in Beijing. This calculation yields the morning and evening peak hour passenger traffic turnover for public transport in Beijing, as shown in Fig. 3.

### Methodological process

**Fig. 4:**
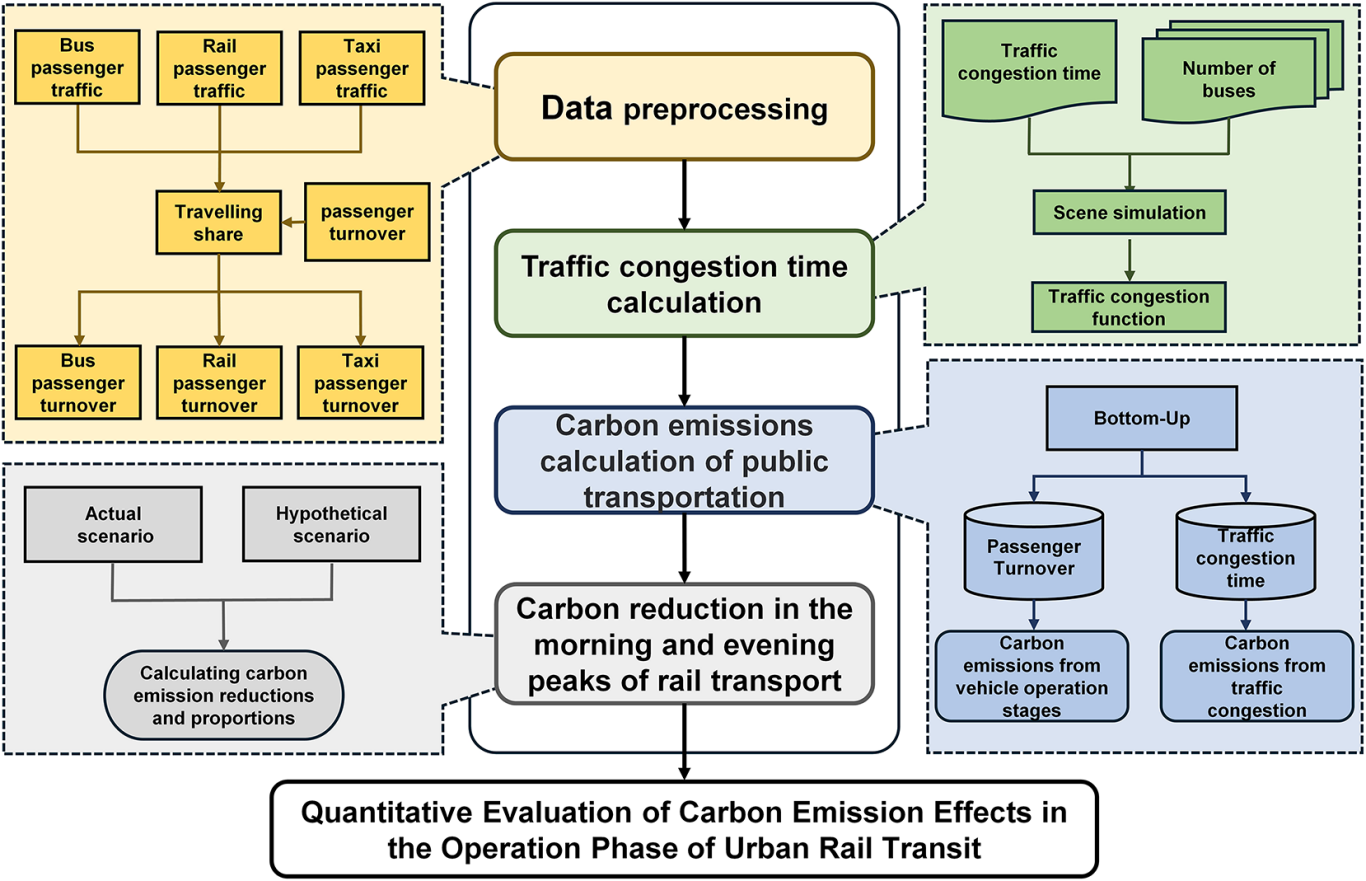
Calculation route of the carbon emission reduction mechanism for urban rail transport.

The initial stage of the study involves preprocessing public transport data to derive the passenger turnover for each mode during the morning and evening peak periods. This is done using the trip share ratio, passenger turnover, and the proportion of trips for each mode during these peak times. Next, we adopted a bottom-up approach to develop a carbon emission calculation model for public transport, using passenger flow and other relevant parameters to estimate traffic congestion times. Finally, we compared carbon emissions during the morning and evening peaks in both the actual operational phase and a hypothetical scenario, calculating the carbon emission reduction and its proportion in urban rail transport. The technical approach is shown in Fig. 4.

#### Traffic congestion state prediction model

The traffic congestion time refers to the duration during which traffic flow on a transportation roadway is hindered, resulting in vehicle delays and trip uncertainty^47^. Metrics used to assess traffic congestion time include average traffic speed, distribution of travel speed, traffic volume, traffic flow patterns, and density, travel time delay, parking duration, queue length at intersections, roadway capacity, and average commute time. Various evaluation systems employ distinct criteria and calculation formulas^48,49^.

The objective of this study is to examine the hypothesis that the absence of urban rail transit leads to a shift in passenger travel patterns, with individuals opting for alternative modes such as buses and taxis. This shift is expected to increase roadway congestion and prolong traffic delays. To investigate the relationship between congestion time, passenger volume, and the number of different transport modes, we first calculated congestion time during the morning and evening peak periods based on the traffic analysis report of major Chinese cities provided by AutoNavi. Our analysis suggests that an increase in passenger traffic for ground transportation leads to longer queuing and congestion times. To alleviate this, congestion time can be reduced by increasing the number of operating vehicles, thereby shortening both queuing and congestion times. Based on passenger capacity calculations and assumptions about the number of transport vehicles in different scenarios, we derived the following formulas for calculating traffic congestion time during the morning and evening peak periods.

Real Scenario:

1 $$t_{{{\text{congestion}}\;{\text{travel}}\;{\text{time}}}} = t_{{{\text{ travel}}\;{\text{time}}}} - \frac{{t_{{{\text{free}}\;{\text{travel}}\;{\text{time}}}} }}{m}$$

where *t*_*congestion travel time*_ represents the traffic congestion time in the real scenario, *t*_*travel time*_ denotes the travel time, *t*_*free travel time*_ refers to the free-flow (unimpeded) travel time, and *m* is the congestion delay index.

Bus Scenario:

2 $${t_{Bus}}=[({P_{r,URT}}+{P_{r,Bus}})/{P_{r,bus}}]*[({N_{b,Bus}}-{N_{r,Bus}})/{N_{r,Bus}}]*{t_r}$$

where *t*_*Bus*_ represents the traffic congestion time in the bus scenario, *P*_*r, URT*_ denotes the urban rail passenger traffic in the real scenario, *P*_*r, Bus*_ refers to the bus passenger traffic in the real scenario, *N*_*b, Bus*_ is the number of buses in the bus scenario, *N*_*r, Bus*_ is the number of buses in the real scenario, and *t*_*r*_ indicates the real congestion time.

Taxi Scenario:

3 $${t_{Taxi}}=[({P_{r,URT}}+{P_{r,Taxi}})/{P_{r,Taxi}}]*[({N_{t,Taxi}}-{N_{r,Taxi}})/{N_{r,Taxi}}]*{t_r}$$

where *t*_*Taxi*_ represents the traffic congestion time in the taxi scenario, *P*_*r, URT*_ denotes the urban rail passenger traffic in the real scenario, *P*_*r, Taxi*_ refers to the taxi passenger traffic in the real scenario, *N*_*t, Taxi*_ is the number of taxis in the taxi scenario, *N*_*r, Taxi*_ is the number of taxis in the real scenario, and *t*_*r*_ indicates the real congestion time.

#### Calculation models for carbon emissions

**Fig. 5:**
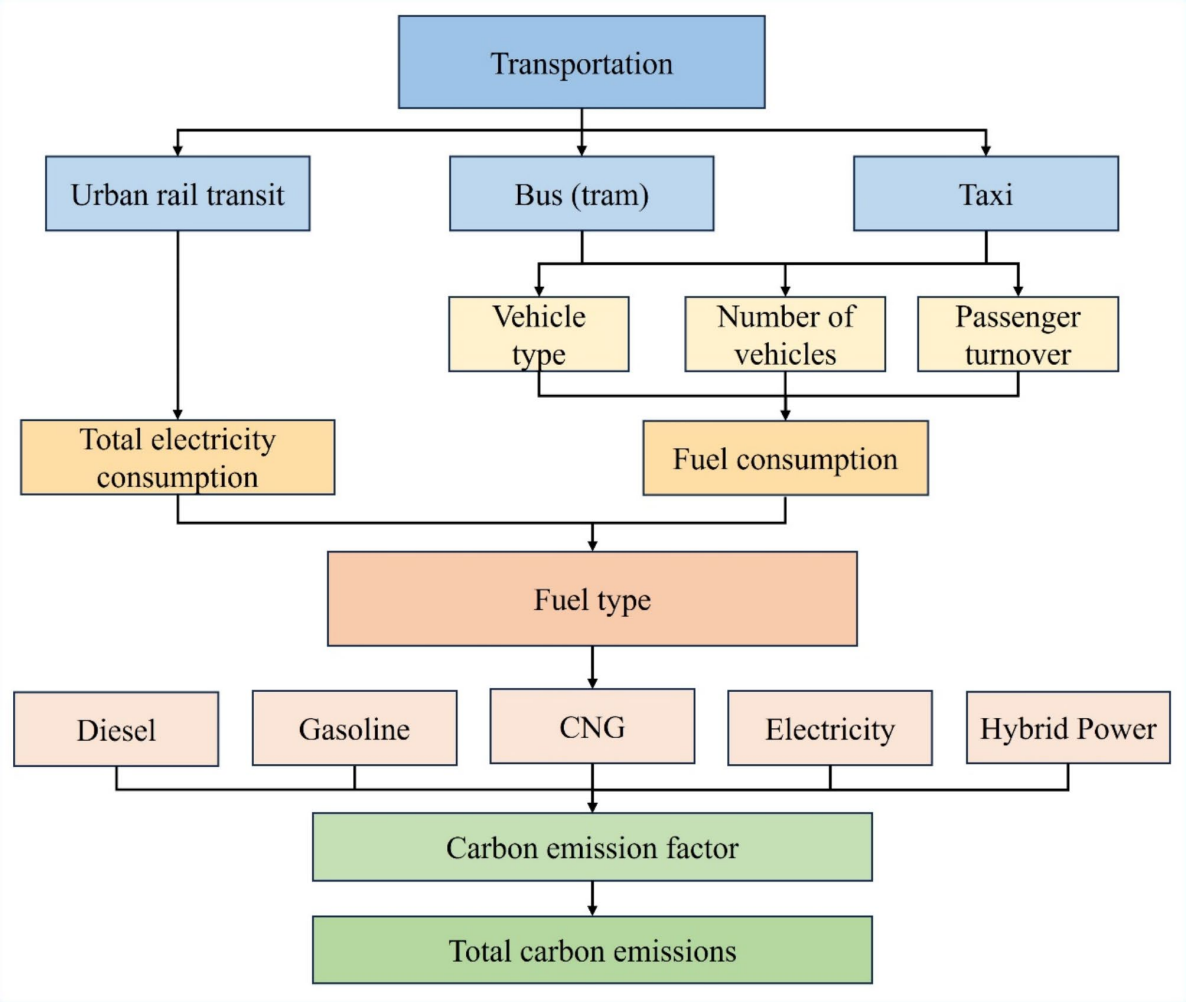
Carbon emission model of urban passenger transport in Beijing.

Carbon emission measurement methodologies are classified into two primary types: ‘top-down’ and ‘bottom-up.’ The ‘top-down’ method derives from macro energy consumption data and estimates emissions based on extensive energy statistics for a specific country or region^50^. This approach is noted for its high accuracy, contingent upon the availability of detailed energy consumption figures. In contrast, the ‘bottom-up’ method is based on field data and empirical observations, providing a practical and adaptable framework for assessing carbon emissions. This method involves directly measuring energy use and operational activities within a particular industry or transportation mode^51,52^. When estimating carbon emissions for urban public transport systems, variables such as traffic congestion, distance covered, and operational time must be taken into consideration. To achieve a more comprehensive and accurate measurement while considering data availability, this study employs two methods to quantify carbon emissions from urban public transport. Figure 5 depicts the carbon emission model for urban passenger transportation in Beijing.

In this study, urban rail transport carbon emissions are quantified using a top-down modelling approach consistent with the National Greenhouse Gas Inventory Guidelines (IPCC, 2013). This method evaluates total electricity consumption and incorporates electricity emission factors, as shown in Eq. (4).

4 $$C_{i}^{t} = \sum\limits_{{t,i}} {EF_{i}^{t} \times E_{i}^{t} }$$

where $\:{\text{C}}_{\text{i}}^{\text{t}}$ represents the carbon emissions from urban rail transport in city i during year t, $\:{\text{EF}}_{\text{i}}^{\text{t}}$ is the electricity emission factor, and $\:{\text{E}}_{\text{i}}^{\text{t}}$ is the electricity consumption of rail transport.

The following formulas are used to assess the environmental impact of buses and taxis. The first formula calculates this impact based on passenger turnover and per capita carbon emissions per kilometre for each mode of transport. The specific formulas are provided below:

5 $${C_{\text{t}}}=\sum\limits_{{{\text{i}}=1}}^{n} {C_{i}^{t}} =\sum\limits_{{i=1}}^{n} {365 \times N_{i}^{t} \times D_{i}^{t} \times M_{i}^{t}}$$

where *C*_*t*_ represents the total emissions from public transport in year t; $\:{\text{C}}_{\text{i}}^{\text{t}}$ represents the carbon emissions of each mode of transport; $\:{\text{N}}_{\text{i}}^{\text{t}}$ represents the average daily travel volume; $\:{\text{D}}_{\text{i}}^{\text{t}}$ represents the average annual travel distance; and $\:{\text{M}}_{\text{i}}^{\text{t}}$represents the per capita carbon emissions per kilometre.

The second objective is to measure carbon emissions based on peak traffic congestion times in the morning and evening, operating speed during peak periods, and energy consumption per unit of time. The calculation formula is as follows:

6 $${C_t}=\sum\limits_{{i=1}}^{n} {C_{i}^{t}} =\sum\limits_{{i=1}}^{n} {Q_{i}^{t}} \times G_{i}^{t} \times T_{i}^{t} \times V_{i}^{t} \times \beta _{i}^{t}$$

where *C*_*t*_ represents the total emissions from public transport in year t, $\:{\text{C}}_{\text{i}}^{\text{t}}$ represents the carbon emissions of each mode of transport, $\:{\text{Q}}_{\text{i}}^{\text{t}}$represents the ownership of each mode of transport, $\:{\text{G}}_{\text{i}}^{\text{t}}$ represents the energy consumption per 100 miles of each mode of transport, $\:{\text{T}}_{\text{i}}^{\text{t}}$ represents the congestion time of each mode of transport during peak hours, $\:{\text{V}}_{\text{i}}^{\text{t}}$ represents the speed of each mode of transport during peak hours, and $\beta _{{\text{i}}}^{{\text{t}}}$ represents the carbon emission factor of each mode of transport.

Given the close relationship between vehicle carbon emissions and driving speed, this study employs a fitting equation for the CO_2_ emission factor in relation to speed, derived from prior research^53^. This equation has been optimized based on the MOVES^54^ vehicle emission model developed by the U.S. Environmental Protection Agency (EPA). It is constructed using the least squares regression analysis method to simulate emissions at various speeds. The CO_2_ emission factor formulas for buses and taxis are presented in Table 3.

**Table 3:** CO_2_ emission factor model.

Vehicle type	CO_2_ emission factor modelling
Bus	$F=0.521+\frac{{16.032}}{v}$
Taxi	$F=0.140+\frac{{3.874}}{V}$

#### Carbon emission reduction modelling in urban rail transits

This study provides a quantitative assessment of the impact of urban rail transport on reducing surface traffic congestion. It develops a model for estimating carbon emissions from urban rail transport, utilising a prediction model for traffic congestion levels and a calculation model for public transport carbon emissions. The carbon emissions reduced by urban rail transit mainly consist of two parts: first, the difference in carbon emissions during the vehicle operation phase before and after the transition; and second, the carbon emissions caused by the additional commuting time due to traffic congestion. The specific calculation formulas are as follows:

7 $$E_{{{\text{reduce}}}} = \Delta E_{{{\text{road}}}} + \Delta E_{{{\text{rail}}}} = \left( {E_{{{\text{h}},{\text{road}}}} - E_{{{\text{s}},{\text{road}}}} } \right) + \left( {E_{{{\text{s}},{\text{rail}}}} - E_{{{\text{h}},{\text{rail}}}} } \right)$$

*E*_*reduce*_ signifies a decrease in carbon emissions within the urban transport system attributable to urban rail transport. *ΔE*_*road*_ indicates the variance in carbon emissions during vehicle operation pre- and post conversion. *ΔE*_*rail*_ represents the carbon emissions attributed to supplementary commuting time induced by traffic congestion. *E*_*s, road*_ and *E*_*h, road*_ denote the carbon emissions of public transport in the standard and hypothetical scenarios, respectively. Similarly, *E*_*s, rail*_ and *E*_*h, rail*_ delineate the carbon emissions stemming from additional commuting time due to traffic congestion in the standard and hypothetical scenarios, respectively.

## Results and analysis

### Analysis of traffic congestion state prediction results

This study incorporates reasonable assumptions regarding the number of transport modes in different scenarios, based on passenger volume, vehicle ownership data from the *Beijing Statistical Yearbook*, and the congestion delay index during morning and evening peak hours as reported in the *Traffic Analysis Report of Major Cities in China* by AutoNavi. Using these assumptions and the proposed formula, the congestion time for each scenario was subsequently calculated.

Under the assumption that urban rail transit is unavailable during the operational phase, this study considers two extreme scenarios. In these scenarios, passengers who would typically travel by rail transit during peak hours shift to surface transportation to meet their travel needs. The first scenario involves passengers exclusively using buses, while the second scenario involves passengers exclusively using taxis. In actual operation, this portion of the passenger flow would be shared between buses and taxis; therefore, the anticipated increase in congestion time is expected to fall between these two scenarios.

**Fig. 6:**
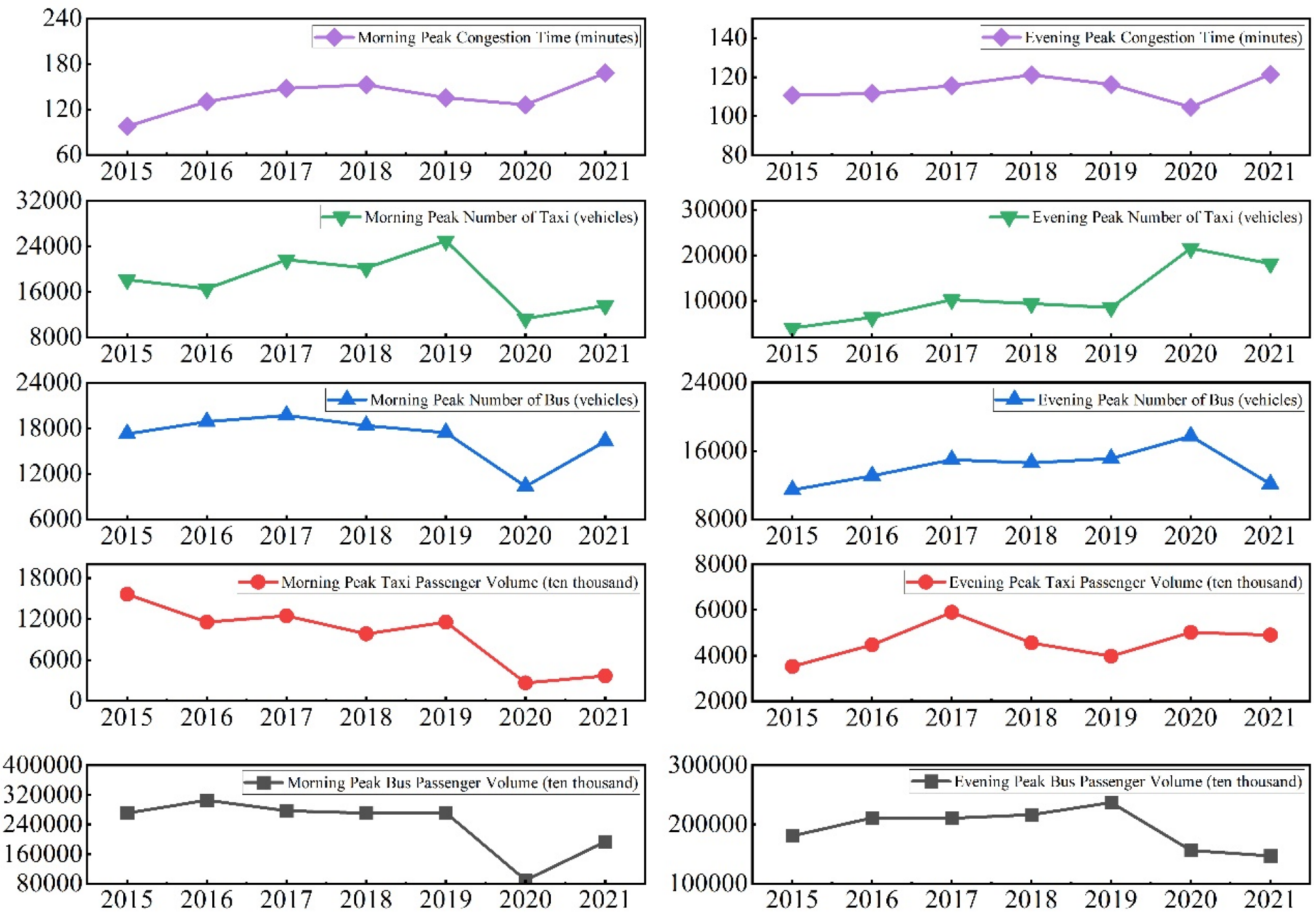
Morning and evening peak congestion times and related data for scenario 1.

*Hypothetical Scenario 1*: Passengers who originally travelled by rail in the morning and evening peak hours return to the ground to take buses to complete their travel needs. For this hypothetical scenario, we have made sensible assumptions regarding the quantity of buses and taxis. The congestion state prediction model is then used to calculate the morning and evening peak periods, and the results are shown in Fig. 6.

**Fig. 7:**
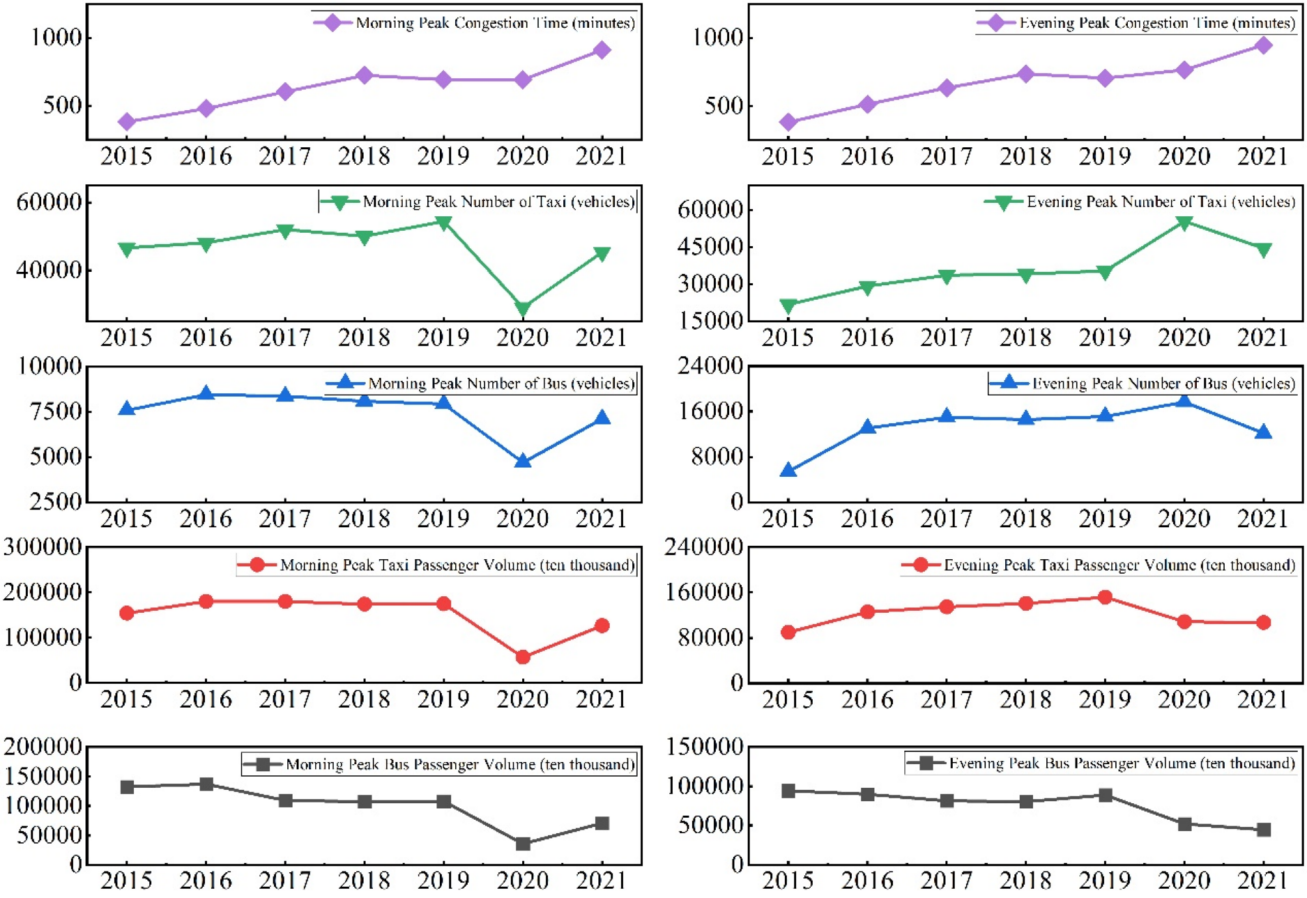
Morning and evening peak congestion times and related data for scenario 2.

*Hypothetical Scenario 2*: Passengers who originally travelled by rail in the morning and evening peak hours return to the ground to take taxis to complete their travel needs. For this hypothetical scenario, we have made sensible assumptions regarding the quantity of buses and taxis. The congestion state prediction model is then used to calculate the morning and evening peak periods, and the results are shown in Fig. 7.

This study analyses the morning and evening peak periods under two extreme hypothetical scenarios and comes to the following conclusions:

1. In Scenario 1, traffic congestion time between 2015 and 2021 exhibits a pattern of increase, followed by decrease, and then another increase. This trend can be explained in two distinct phases. First, from 2015 to 2018, the substantial growth in rail transport prompted some passengers to switch back to road transport, resulting in increased road congestion and prolonged congestion times. Second, from 2019 to 2020, preventive measures implemented in response to the Xinguang epidemic regulated surface transport, significantly reducing congestion times. By 2021, with China’s anti-epidemic measures restoring public transport to normal levels, congestion times began to rise again.In contrast, under Scenario 2, congestion time remains consistently higher due to the shift of rail passengers to surface transport, such as taxis. This shift exacerbates traffic pressure and vehicle density, leading to significant increases in congestion time during morning and evening peak periods. Congestion time in this scenario exceeds 300 min during peak periods—an extreme level far beyond the reasonable range for real-world traffic operations. While this outcome holds theoretical value for simulations, it is impractical for real-world application.
2. Comparison of Morning and Evening Peaks: In Hypothetical Scenario 1, morning peak congestion time is significantly longer than evening peak congestion time, with a difference of approximately 30 min. This discrepancy arises because passenger flow for rail transport is substantially higher in the morning peak than in the evening peak. When some rail passengers shift to ground transport, the resulting congestion imposes greater pressure on the road network during the morning peak. In Scenario 2, the difference between morning and evening peak congestion times is less pronounced, with both periods experiencing severe congestion. This reflects the persistently high traffic pressure on surface roads due to the absence of rail transport. Moreover, overall congestion time in Scenario 2 far exceeds the actual operational values, underscoring the critical role of rail transport in alleviating surface traffic burdens and its significant impact on urban traffic management.

### Analysing the carbon emissions calculations

The annual carbon emission factors for regional power grids used in this study are obtained from data published by the Ministry of Ecology and Environment of the People’s Republic of China. Additionally, considering related studies^42,55^ and the specific characteristics of transportation in Beijing, the carbon emission factors for various fuel types used by buses and taxis in the city were determined. To ensure scientific rigor and calculation accuracy, this paper categorizes the carbon emission factors by fuel type and adjusts them according to actual traffic conditions and fuel usage. Table 4 provides detailed carbon emission factor data.

**Table 4:** Carbon emission factors of different traffic types and fuel types.

Vehicle type	Fuel type	Carbon emission factor
Bus	Diesel	0.0466 kg/(person*km)
	CNG	0.0156 kg/(person*km)
	Electricity	0.0122 kg/(person*km)
	Hybrid power	0.0174 kg/(person*km)
Taxi	Gasoline	0.2612 kg/(person*km)
	CNG	0.1232 kg/(person*km)
	Electricity	0.1123 kg/(person*km)
	Hybrid power	0.1362 kg/(person*km)

Based on the data mentioned above, this study evaluates the carbon emissions of Beijing’s public transport system under real-world operational scenarios from 2015 to 2021. The results are summarized in Table 5.

**Table 5:** Estimated carbon emissions from public transport in Beijing, 2015–2021 (ten thousand tons).

Year	Public Trams	Urban rail transit	Taxi	Total
2015	118.3350	73.1324	120.3341	311.8014
2016	105.4666	85.0614	94.1967	284.7246
2017	85.4880	90.7128	72.8129	249.0137
2018	78.0503	85.9314	61.3111	225.2929
2019	73.6612	93.9503	58.9116	226.5232
2020	40.6905	43.7779	31.3570	115.8254
2021	47.3030	48.8499	37.1111	133.2639

The data presented above reveal significant temporal trends in the carbon emissions of different transport modes. From 2015 to 2020, bus carbon emissions consistently decreased, from 1,183,350 tonnes to 406,905 tonnes. In contrast, urban rail transit emissions increased from 731,324 tonnes in 2015 to 939,503 tonnes in 2019, before declining due to the impact of the epidemic. Although taxi emissions remain relatively low, their contribution to total emissions is not negligible. Overall, the carbon emissions of Beijing’s public transport system declined from 2015 to 2018, stabilized in 2019, and dropped further in 2020 due to Covid-19-related restrictions. Emissions rebounded in 2021 as travel restrictions were lifted and traffic resumed.

**Fig. 8:**
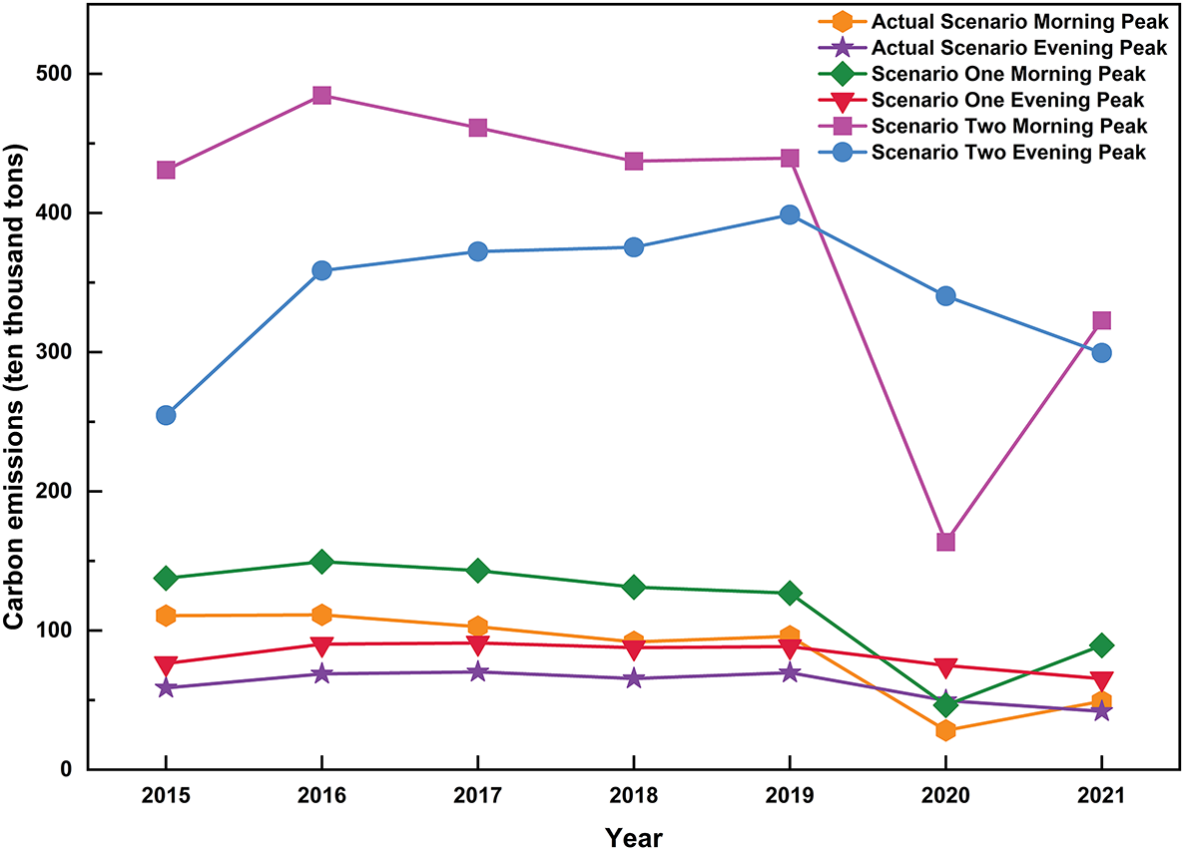
Carbon emissions of Beijing public transport in morning and evening peaks under different scenarios.

The energy-saving and emission reduction effects of urban rail transport, as well as its ability to alleviate traffic congestion, depend on the spatial and temporal distribution of passenger flows. Therefore, this study uses the model described above to calculate the morning and evening peak carbon emissions of Beijing’s public transport under Scenarios 1 and 2. To improve the accuracy of the results, the maximum congestion time is defined when estimating the carbon emissions for these hypothetical scenarios. The results show that carbon emissions in Scenario 2 are significantly higher than in Scenario 1, with both exceeding those of the actual operational phase. Notably, during the morning peak, carbon emissions increased significantly compared to the evening peak. The complete results are shown in Fig. 8.

### Analysis of the results of carbon emission reduction in urban rail transport

This study utilises the aforementioned traffic congestion state prediction model and carbon emission calculation model to quantitatively evaluate the carbon emissions during the morning and evening peak periods from 2015 to 2021. This is achieved by converting the urban rail traffic flow to bus and taxi traffic, while considering the spatial and temporal distribution characteristics of passenger flow.

**Fig. 9:**
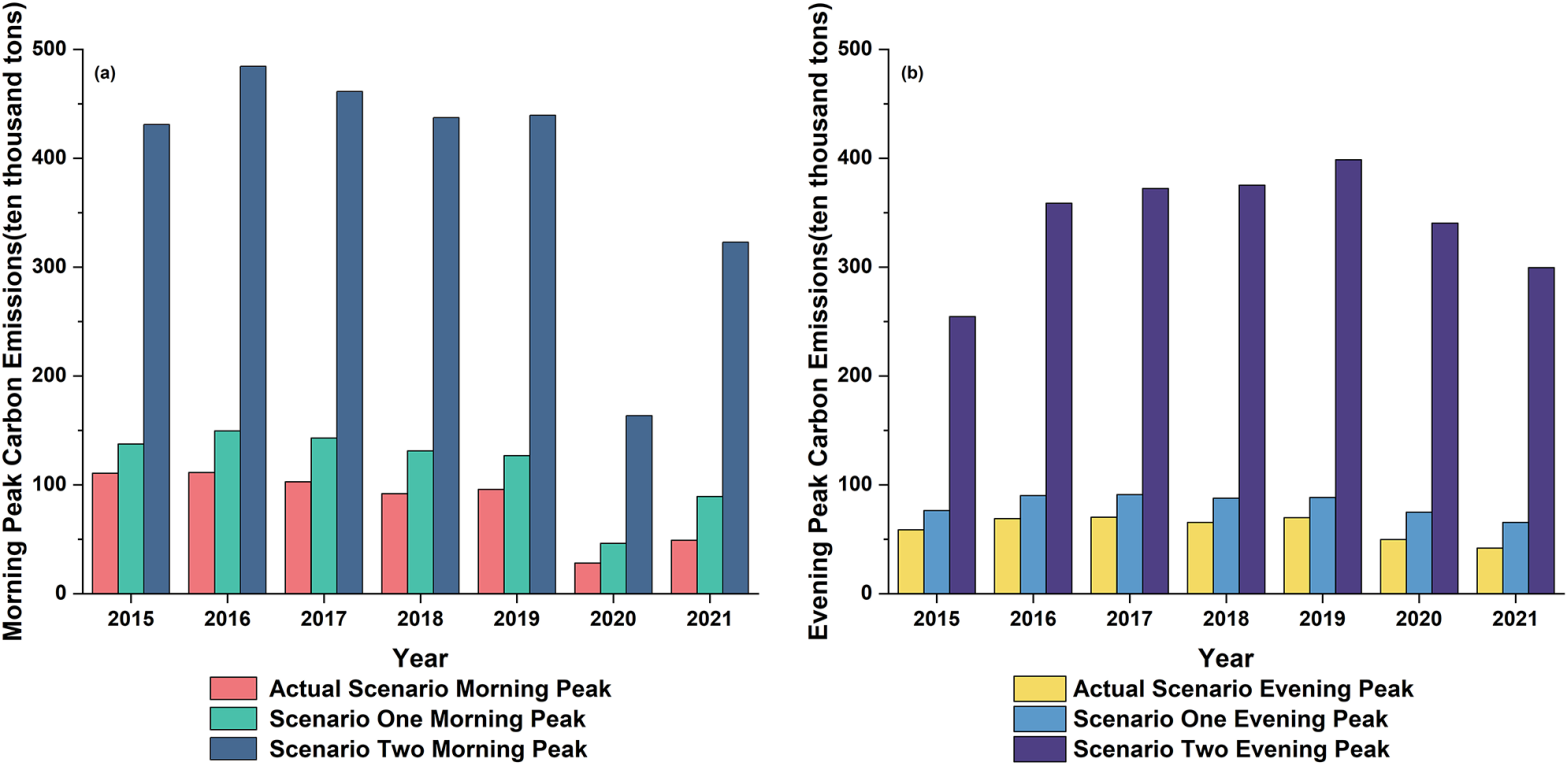
Morning and evening peak carbon emissions in Beijing under the different scenarios.

Initially, we calculated the carbon emissions of Beijing’s public transportation system during actual peak operational hours. Subsequently, we hypothesized various public transportation parameters for different scenarios, calculating the carbon emissions for each hypothetical situation and the emissions resulting from the conversion of travel modes leading to traffic congestion. Finally, we computed the differences in emission levels between the actual operational phase and the hypothetical scenarios. The specific results are illustrated in Fig. 9.

In hypothetical scenario 1, we assume that passengers originally using urban rail transit during the operational phase switch to buses, and we quantitatively evaluate the impact on urban carbon emissions. The results indicate that if passengers choose buses during peak hours, carbon emissions would increase by 24–82% in the morning and 27–56% in the evening. This increase is likely due to the lower capacity and efficiency of buses during peak times compared to urban rail transit, making it difficult to meet passenger demand, as well as their higher carbon emission levels, which contribute to increased traffic congestion and carbon emissions.

In hypothetical scenario 2, assuming all urban rail transit passenger flow switches to taxis, we quantitatively evaluate the impact of this change on carbon emissions. The results show that the use of taxis leads to an increase in carbon emissions during peak hours by 289–556% in the morning and 333–614% in the evening, indicating a significant rise in carbon emissions. This is because taxis become less efficient during traffic congestion, and their per-vehicle emissions are higher.

In both scenarios, carbon emissions are significantly higher than in the actual operation scenario, highlighting the crucial role of urban rail transit in reducing carbon emissions within Beijing’s public transport system. Carbon emissions in Scenario 2 are several times higher than in the standard scenario, suggesting that converting all rail passengers to taxi passengers would substantially raise carbon emissions, potentially causing severe gridlock in the road transport system. In contrast, the relatively lower carbon emissions in Hypothetical Scenario 1 indicate that buses produce fewer emissions than taxis and can help alleviate traffic congestion and reduce carbon emissions to some extent. However, the study’s findings show that even buses cannot achieve the same level of energy savings and emission reductions as urban rail transit. This underscores the need to enhance the service capacity and coverage of urban rail transit for the sustainable development of Beijing’s public transport system.

## Conclusions and disscussions

### Conclusions

This paper focuses on the operational phase of Beijing’s urban rail transit, selecting the city’s peak morning and evening passenger flow as the subject of study. The period from 2015 to 2021, encompassing the time before and after the COVID-19 pandemic, is chosen as the timeframe for analysis, with the assumption that all original rail transit passenger flow is converted to bus and taxi flow during this period. By successively establishing a traffic congestion prediction model, a carbon emission calculation model, and an urban rail transit carbon reduction model, the paper quantitatively analyzes the role of urban rail transit in alleviating ground traffic congestion and reducing carbon emissions. The following conclusions are drawn:

1. During the morning and evening rush hours, if the entirety of the original rail transit passenger flow is redirected to bus or taxi services, the congestion time will exceed the actual operating time considerably. This will render the definition of morning and evening peak meaningless, affect subsequent passenger transport, and cause Beijing’s road traffic network to become severely congested. It is evident that urban rail transit plays a pivotal role in mitigating ground traffic congestion.
2. The decline in carbon emissions from Beijing’s public transport system between 2015 and 2018 reflects two key trends: the growth in urban transport demand and the optimization of transport modes. The rapid expansion of rail infrastructure significantly reduced passenger reliance on buses and taxis. However, during 2020 and 2021, carbon emissions decreased substantially due to the global COVID-19 pandemic. This reduction can be attributed to travel restrictions, the increased adoption of teleworking, and decreased public transportation usage.
3. If the current urban rail transit passenger flow is entirely shifted to buses and taxis, a comprehensive calculation of carbon emissions from ground traffic congestion, increased carbon emissions from ground transportation vehicles, and reduced emissions due to the cessation of operational rail transit reveals that the increase in carbon emissions during peak morning and evening hours would be 24-82% and 289-614%, respectively. This conclusively demonstrates the significant contribution of operational urban rail transit projects in reducing urban traffic carbon emissions.
4. When assessing the carbon emission impact of urban rail transit, it is crucial to differentiate between operational lines and planned (new) lines. The assessment of operational lines should consider current passenger flow, operational efficiency, and energy consumption patterns. For planned lines, the focus should be on the resources invested during the construction phase, potential operational models, and the expected passenger appeal. Only through this phased approach can more accurate and comprehensive results be obtained.

### Proposed policy recommendations

On the basis of the key findings of this study, we would like to propose the following policy recommendations:

1. Green urban rail transport projects involve measures such as strengthening green planning and design, optimizing rail line layouts, adopting energy-saving technologies, and minimizing unnecessary energy consumption. Enhanced passenger flow forecasting, improved scheduling management, and reasonable train frequency and departure interval arrangements can reduce idling rates and improve operational efficiency. Additionally, integrating urban rail transit with walking and cycling promotes sustainable travel. Photovoltaic power generation facilities installed in stations, depots, and other locations harness solar energy, reducing traditional energy consumption and maximizing the use of clean energy.
2. To increase the full-load rate of trains during morning and evening peak hours, train frequency should be increased, and departure intervals shortened. These measures will reduce passenger waiting time and lower carbon emissions per passenger. Additionally, promoting carpooling services, especially in the taxi sector, can further decrease emissions. Advanced technology should be employed to match passengers with drivers accurately, minimizing single-passenger trips and reducing per capita carbon emissions. Furthermore, dedicated bus lanes and taxi parking areas should be established to enhance the efficiency of public transport operations. These measures will encourage more individuals to choose environmentally friendly and convenient public transportation options.

### Limitations analysis

The methodology and data employed in this study have certain limitations. Firstly, due to the lack of specific route data, the analysis was not conducted on any particular line or combination of lines; instead, it was approached from a macro perspective, examining the outcomes of urban rail transit in alleviating traffic congestion and reducing carbon emissions. Future research should aim for a finer granularity. Secondly, for the parameters used in fitting the traffic congestion time prediction model, the impact of related parameters such as driving speed and load factor should be further considered. These improvements will aid in a more in-depth evaluation of the impact of urban rail transit on easing traffic congestion and reducing carbon emissions.

## Data Availability

Datasets will be available upon request, contact Luzhou Lin at: linluzhou@bfsu.edu.cn.
